# High Content Imaging and Analysis Enable Quantitative *In Situ* Assessment of CYP3A4 Using Cryopreserved Differentiated HepaRG Cells

**DOI:** 10.1155/2014/291054

**Published:** 2014-09-08

**Authors:** Aarati R. Ranade, Melinda S. Wilson, Amy M. McClanahan, Andrew J. Ball

**Affiliations:** ^1^Molecular and Cell Biology Systems, EMD Millipore Corporation, 290 Concord Road, Billerica, MA 01821, USA; ^2^Donald Danforth Plant Science Center, 975 North Warson Road, St. Louis, MO 63132, USA; ^3^Barnes-Jewish Hospital, One Barnes-Jewish Hospital Plaza, St. Louis, MO 63110, USA

## Abstract

High-throughput imaging-based hepatotoxicity studies capable of analyzing individual cells *in situ* hold enormous promise for drug safety testing but are frequently limited by a lack of sufficient metabolically competent human cells. This study examined cryopreserved HepaRG cells, a human liver cell line which differentiates into both hepatocytes and biliary epithelial cells, to determine if these cells may represent a suitable metabolically competent cellular model for novel High Content Analysis (HCA) applications. Characterization studies showed that these cells retain many features characteristic of primary human hepatocytes and display significant CYP3A4 and CYP1A2 induction, unlike the HepG2 cell line commonly utilized for HCA studies. Furthermore, this study demonstrates that CYP3A4 induction can be quantified via a simple image analysis-based method, using HepaRG cells as a model system. Additionally, data demonstrate that the hepatocyte and biliary epithelial subpopulations characteristic of HepaRG cultures can be separated during analysis simply on the basis of nuclear size measurements. Proof of concept studies with fluorescent cell function reagents indicated that further multiparametric image-based assessment is achievable with HepaRG. In summary, image-based screening of metabolically competent human hepatocyte models cells such as HepaRG offers novel approaches for hepatotoxicity assessment and improved drug screening tools.

## 1. Introduction

Drug-induced hepatotoxicity is a major contributor to the high attrition rates of drug candidates during preclinical and clinical drug development [1]. It is also responsible for many postlaunch withdrawals and labeling restrictions for drugs which have successfully gone through the discovery and development process [2]. Assessment of hepatotoxicity remains difficult because of challenges associated with* in vivo* models [3] and the high cost and limited availability of liver tissue for* in vitro* studies [4]. Current* in vitro* models for assessing hepatotoxicity are limited by (a) scarcity, variability, and short life span in culture of primary human hepatocytes [4]; (b) lack of metabolic activity in widely used liver cell lines such as HepG2 [5]; and (c) the complex long-term protocols required to differentiate progenitor cells [6].

In recent years, HepaRG cells have emerged and are being increasingly adopted as an alternative to HepG2 cells and primary hepatocytes for* in vitro* hepatotoxicity studies, overcoming many of the limitations associated with existing hepatocyte cellular models [7]. The HepaRG human cell line was established from a tumor of a female patient suffering from chronic hepatitis C infection and hepatocarcinoma [8]. When passaged at low density, they are able to recover and differentiate into both hepatocytes and biliary epithelial cells and are thus considered to be progenitor cells [9]. Gene expression profiling has shown that HepaRG cells are remarkably close to human hepatocyte populations [10]. Unlike other immortal hepatic cell lines such as HepG2, HepaRG display many characteristics of primary human hepatocytes, including cytochrome P450 mediated metabolism, transporter functions, and expression of key nuclear receptors known to play important role in liver function following drug exposure [11]. Accordingly, these cells have served as an effective surrogate for primary human hepatocytes in a wide variety of liver-specific functional assays [7, 11–13]. Initially, HepaRG cells required several weeks of culture to bring them to a differentiated state; however, HepaRG cells have recently become available in a ready-to-use cryopreserved differentiated format which has shown promise for drug metabolism studies [14].

High Content Analysis (HCA), an imaging-based quantitative cellular analysis technology, enables multiparametric detection of events in individual cells* in situ* and is well-suited for high-throughput assessment of hepatotoxicity [15]. Pioneering work has extensively validated this technique for analysis of HepG2 cells and primary hepatocytes [16–19].

This study aimed to characterize the cryopreserved differentiated HepaRG cells for use as human hepatocyte surrogates in High Content Analysis applications and to determine if imaging-based detection of CYP3A4 activity is feasible. Specific goals were (a) to determine if cryopreserved differentiated HepaRG cells retain key functional hepatocyte characteristics, (b) to determine if these cells are amenable to multiparametric HCA under conditions where CYP3A4 activity is retained, and (c) to determine optimal assay conditions for the application of these cells to imaging-based CYP3A4 expression studies and multiparametric hepatotoxicity assessment.

## 2. Materials and Methods

### 2.1. Reagents

Cryopreserved HepaRG cells (Catalog # MMHPR116), HepaRG thawing/plating medium supplement (Catalog # MMADD671), HepaRG induction medium supplement (Catalog # MMADD641), and HepaRG culture medium supplement (Catalog # MMADD621) were from EMD Millipore (Billerica, MA). Williams E Medium (WEM) and GlutaMAX were purchased from* In Vitro *Technologies, Inc. (Baltimore, MD). HepG2 cells (Catalog # HB-8065) were obtained from ATCC (Manassas, VA). For HepG2 culture, MEM/EBSS (Catalog # SH3024401), fetal bovine serum (Catalog # SH3007103), nonessential amino acids (Catalog # SH3023801), sodium pyruvate (Catalog # SH3023901), and penicillin/streptomycin/glutamine (Catalog # SV3008201) were obtained from Thermo Fisher Scientific, Inc. (Waltham, MA). 96-well polystyrene tissue culture plates and rat tail collagen type I were obtained from BD Biosciences (San Jose, CA). Dimethyl sulfoxide (DMSO), Hoechst 33342 nuclear stain, formaldehyde, Triton X-100, Periodic Acid Schiff (PAS) reagents, rifampicin (RIF), and omeprazole (OME) were purchased from Sigma-Aldrich (St. Louis, MO). ELISA kits for detection of albumin and alpha-1-antitrypsin were purchased from Bethyl Laboratories, Inc. (Montgomery, TX). GSH-Glo glutathione and P450-Glo assay kits were purchased from Promega Corporation (Madison, WI). Rabbit anti-human cytochrome P450 enzyme CYP3A4 polyclonal antibody (Catalog # AB1254), mouse anti-Cytokeratin 19 monoclonal antibody (Catalog # MAB3238), donkey anti-rabbit IgG antibody, Cy3 conjugate (Catalog # AP182C), and donkey anti-mouse IgG antibody, FITC conjugate (Catalog # AP192F), were from EMD Millipore (Billerica, MA). MitoTracker Green FM (Catalog # M-7514), Monochlorobimane (mBCI) (Catalog # M-1381MP), CellTracker Red CMTPX (Catalog # C34552), Tetramethylrhodamine, Methyl Ester, Perchlorate (TMRM) (Catalog # T668), and Alexa Fluor 568 Phalloidin (Catalog # A12380) were obtained from Life Technologies (Carlsbad, CA). All other materials were from EMD Millipore (Billerica, MA).

### 2.2. Cell Culture

Cryopreserved HepaRG cells were thawed and cultured in collagen coated 96-well plates according to the supplier's protocol. HepaRG cells were allowed to attach for 6 hrs, at which time the HepaRG thawing/plating medium was renewed. The cells were maintained in HepaRG culture medium which was renewed every day. HepG2 cells were thawed and cultured according to vendor's instructions with media replenishment 1 day after thaw and every 3 days thereafter.

### 2.3. Assessment of Hepatocyte-Associated Functional Activity

Following 48 h culture after thaw, HepaRG and HepG2 cells cultured in 96-well plates at a seeding density of 50,000 cells per well were assessed for a panel of functional activities associated with human hepatocytes, using well-established reagents and protocols. Periodic Acid-Schiff staining was performed according to manufacturer's instructions in order to identify the presence of glycogen within the cell cultures. Secretion of albumin and alpha-1-antitrypsin by HepG2 and HepaRG cells was assessed using ELISA kits for each, with tests performed according to manufacturer's instructions, using albumin and alpha-1-antitrypsin standards provided with the test kits to enable quantitation of each in control samples of tissue culture media which had not been in contact with cells, or in tissue culture media aspirated from HepG2 or HepaRG cell cultures following 48 h culture after thaw. Levels of glutathione (GSH) in HepG2 and HepaRG and its depletion in response to treatment with buthionine sulfoximine (BSO) were assessed using a luminescence-based assay performed according to manufacturer's instructions for adherent cell cultures, whereby tissue culture medium is removed from the cells prior to incubation of the cells with luminescent detection reagents.

### 2.4. Determination of Cytochrome P450 CYP3A4 Activity

Basal levels of cytochrome P450 (CYP3A4) enzyme activity were measured after culturing the cryopreserved HepaRG or HepG2 cells for 3 days. For induction studies, HepaRG and HepG2 cells were exposed to the prototypical CYP3A4 inducer rifampicin (RIF; 10 uM) for 72 hrs, starting the induction on Day 3 after plating the cells. DMSO (0.1%) was used as a vehicle control. Basal and induced CYP3A4 activities were measured using P450-Glo Assays, according to the manufacturer's instructions. Induced CYP3A4 enzyme activity was calculated as fold increase over DMSO controls.

### 2.5. Cell Staining

For multiplexed Hoechst/CK-19/CYP3A4 staining, at the end of the induction period, plates were removed from the incubator and the cells were immediately fixed with 100 *μ*L of prewarmed fixation solution containing 7.4% formaldehyde for 30 min at room temperature. Following fixation, each well was gently washed twice with a cell permeabilization buffer containing 0.25% Triton X-100. A primary antibody working solution containing rabbit anti-CYP3A4 (1 : 1000 dilution) and mouse anti-Cytokeratin-19 (1 : 500 dilution) antibodies was prepared in this buffer and 50 *μ*L was applied to each well; then plates were incubated for 1 h at room temperature. Following primary antibody incubation, cells were washed three times with PBS. A secondary antibody working solution containing Cy3-donkey anti-rabbit antibody (5 *μ*g/mL), FITC-donkey anti-mouse antibody (5 *μ*g/mL), and Hoechst 33342 nuclear stain (10 *μ*g/mL) was prepared and 50 *μ*L was applied to each well for 1 h at room temperature. Cells were then washed twice with PBS, and plates were then stored, sealed, and protected from light at 4°C until image acquisition.

For other stainings, MitoTracker Green FM was added to live, unfixed HepaRG cells at a concentration of 0.1 *μ*M and incubated for 30 minutes prior to imaging; mBCI dye was added to live, unfixed HepaRG cells at a concentration of 80 *μ*M and incubated for 10 minutes prior to imaging; CellTracker Red CMTPX was added to live, unfixed HepaRG cells at a concentration of 0.5 *μ*M and incubated for 30 minutes prior to imaging; TMRM dye was added to live, unfixed HepaRG cells at a concentration of 0.1 *μ*M and incubated for 30 minutes prior to imaging; Alexa Fluor 568 Phalloidin was added to fixed HepaRG cells at a concentration of 0.165 *μ*M and incubated for 60 minutes prior to imaging.

## 3. High Content Analysis

Assay plates were imaged with a GE IN Cell Analyzer 1000 high content imaging system and images were analyzed with GE IN Cell Analyzer 1000 Workstation (3.7) software, utilizing the multitarget analysis algorithm to segment cellular features based on size and fluorescence intensity-related criteria. Imaging was performed using a 20x objective lens, acquiring 10 fields of view for each wavelength per well. Image analysis parameters were optimized in GE Workstation (3.7) software for accurate and robust cell and feature segmentation, using representative images of treated and untreated cells. Automated image analysis was performed on each well, collecting data on a cell-by-cell basis, which was then averaged within the application, and the reported well-by-well summary was used for subsequent analysis.

### 3.1. Statistical Analysis

For all assays, biological replicates from three separate wells were averaged to obtain the mean and standard error of the mean for each treatment dose. To determine drug-induced cellular changes, comparisons were made between each dose and vehicle-only controls. Student's* t*-test (two-tailed distribution, two-sample equal variance, *P* < 0.05) was used to determine the significance of responses. GraphPad Prism software was used to generate all graphs.

## 4. Results and Discussion

HepaRG cells represent an attractive option for hepatotoxicity applications because they retain many features of primary human hepatocytes which are not present in other hepatic cell lines [12], including activity of the critically important drug metabolizing enzyme CYP3A4 [20]. This study characterized the cryopreserved differentiated form of the cells and compared them to the widely used HepG2 cell line with regard to phenotypic features characteristic of human hepatocytes. While High Content Analysis represents a powerful technique for hepatotoxicity and drug screening applications [16–19, 21, 22], the quality of HCA data is largely determined by the effectiveness of image analysis algorithms at accurately identifying and segmenting cellular features of interest. Since HepaRG differentiate into a complex mix of hepatocyte and biliary epithelial cells [9] and since established protocols for culture of HepaRG cells generally require relatively high cell seeding densities which may confound image segmentation algorithms, we hoped to establish an optimal set of conditions for HepaRG culture which would enable quantitative HCA without compromising CYP3A4 activity in these cells.

**Figure 1:**
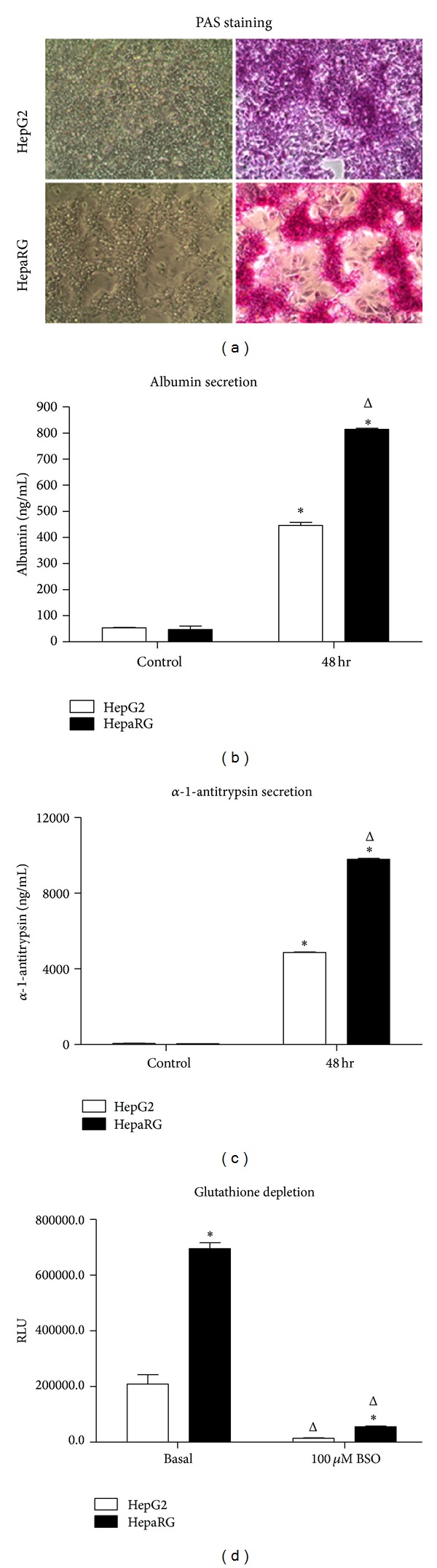
Expression of glycogen, albumin, alpha-1-antitrypsin, and glutathione in HepaRG and HepG2 cells seeded at 50,000 cells per well in 96-well plates and cultured for 48 hrs following thaw. (a) Periodic Acid-Schiff staining for glycogen in HepG2 (upper panels) and HepaRG (lower panels). Unstained 10x phase contrast images are shown on the left side; 10x images of cells stained with PAS reagents are shown on the right side. (b) Secretion of albumin into tissue culture medium by HepaRG and HepG2 cells. Data represent mean ± SEM for 3 independent experiments. ∗ represents *P* < 0.05 versus control measurements taken from culture media which had been unexposed to cells; Δ represents *P* < 0.05 versus HepG2 cells. (c) Secretion of alpha-1-antitrypsin into tissue culture medium by HepaRG and HepG2 cells. Data represent mean ± SEM for 3 independent experiments. ∗ represents *P* < 0.05 versus control measurements taken from culture media which had been unexposed to cells; Δ represents *P* < 0.05 versus HepG2 cells. (d) Levels of glutathione in untreated cells and in HepaRG and HepG2 cells treated for 12 hrs with 100 *μ*M buthionine sulfoximine (BSO). Data represent mean ± SEM for 3 independent experiments. ∗ represents *P* < 0.05 versus HepG2 cells; Δ represents *P* < 0.05 versus cells not treated with BSO.

We first characterized cultured cryopreserved differentiated HepaRG with regard to a panel of functional markers associated with primary human hepatocytes, comparing HepaRG with HepG2 cells (Figure 1). Using a Periodic Acid-Schiff (PAS) staining system with a hematoxylin nuclear counter stain, PAS positive substances stain pink and nuclei are blue. The normal liver contains a large amount of glycogen, leading to hepatocytes staining intensely pink with a PAS stain.  Figure 1(a) shows that HepaRG cells stain strongly pink with PAS staining, indicative of high glycogen content, whereas HepG2 cells stain much less strongly for glycogen, with the blue nuclear counter stain dominating (Figure 1(a)). Albumin secretion is characteristic of hepatocytes, and both HepaRG and HepG2 cells were found to secrete significant amounts of albumin into tissue culture medium (Figure 1(b)), although at equivalent cell seeding densities HepaRG were observed to secrete significantly more albumin than HepG2 cells (1.8-fold greater than HepG2; *P* > 0.05; Figure 1(b)). Healthy hepatocytes also secrete and export the protease inhibitor alpha-1-antitrypsin. Similar to albumin, both HepaRG and HepG2 cells released significant amounts of alpha-1-antitrypsin into tissue culture medium (Figure 1(c)), with HepaRG releasing significantly more than HepG2 cells seeded at an equal density (2.0-fold greater than HepG2; *P* > 0.05; Figure 1(c)). Hepatocytes also synthesize glutathione, an important cellular feature involved in regulating cellular metabolism and responses to several types of toxic challenge. Both HepaRG and HepG2 cells synthesize glutathione (Figure 1(d)) although untreated HepaRG cells were found to synthesize significantly more glutathione than the equivalent number of HepG2 cells (*P* > 0.05; Figure 1(d)). In both HepaRG and HepG2 cells, glutathione synthesis was significantly depleted (*P* > 0.05; Figure 1(d)) by treatment with buthionine sulfoxide (BSO). Taken together, these data suggested that cryopreserved differentiated HepaRG cells, more so than HepG2, exhibit multiple phenotypic characteristics associated with primary hepatocytes. However, for drug safety and hepatotoxicity applications, of greater importance is cellular metabolic function, particularly activity of CYP3A4 and other enzymes associated with drug metabolism [23].

**Figure 2:**
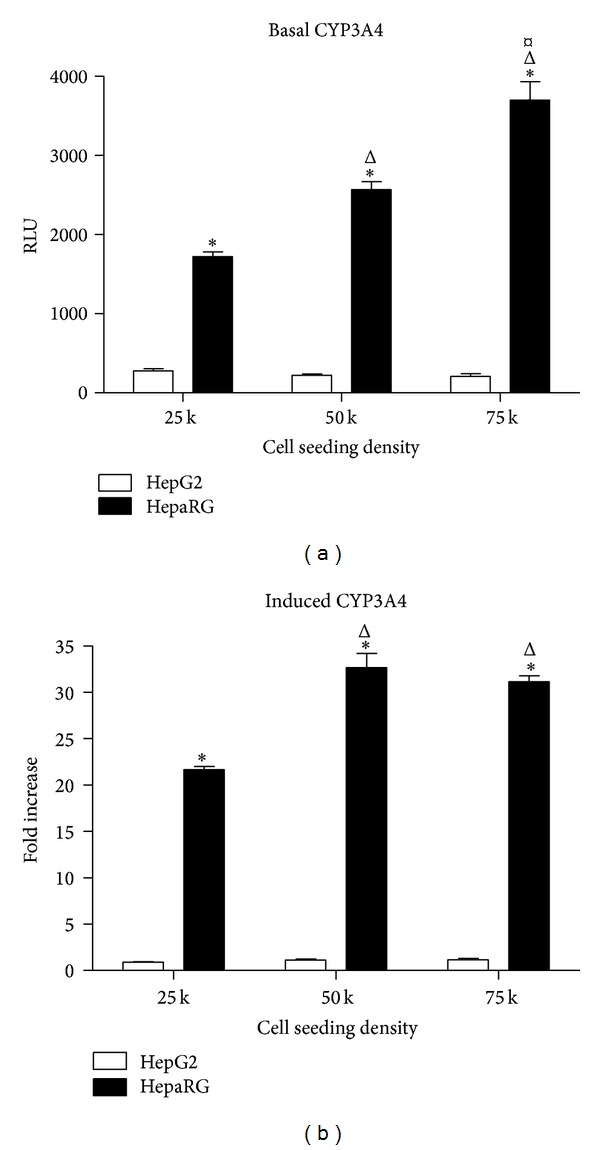
Basal and induced CYP3A4 activity in HepaRG and HepG2 cells. (a) Basal CYP3A4 activity of HepaRG and HepG2 cells plated in 96-well collagen-coated plates at seeding densities of 25,000, 50,000, and 75,000 cells per well and cultured for 72 hours. Data represent mean ± SEM for 3 independent experiments. ∗ represents *P* < 0.05 versus HepG2 cells at the same seeding density; Δ represents *P* < 0.05 versus HepaRG cells seeded at 25,000 cells per well; *¤*  represents *P* < 0.05 versus HepaRG cells seeded at 50,000 cells per well. (b) CYP3A4 induction in HepaRG and HepG2 cells plated in 96-well plates at seeding densities of 25,000, 50,000, and 75,000 cells per well and treated with DMSO (0.1%) or rifampicin (RIF, 10 *μ*M) for 72 hrs starting at Day 3 in culture. Data represent mean ± SEM for 3 independent experiments. ∗ represents *P* < 0.05 versus HepG2 cells at the same seeding density; Δ represents *P* < 0.05 versus HepaRG cells seeded at 25,000 cells per well.

As the primary drug metabolizing enzyme isoform in humans [23], expression and activity of CYP3A4 in cellular hepatocyte models are highly desirable. We first determined the effect of cell seeding densities on basal CYP3A4 enzyme activity in HepaRG, comparing these cells with HepG2 cells (Figure 2(a)). For this, cells were plated at three different seeding densities: 25,000, 50,000, and 75,000 cells per well in 96-well plates. The basal CYP3A4 activity was determined on Day 3 after thawing and plating the cells. In accordance with previous observations [24], basal activity of CYP3A4 in HepG2 cells was extremely low, reflecting a well-known limitation of this cell type. At all three seeding densities tested, HepaRG cells exhibited significantly greater basal CYP3A4 activity than HepG2 cells (Figure 2(a)). In HepaRG cells, basal CYP3A4 activity appeared to be closely linked to cell seeding density, with basal CYP3A4 activities in these cells increasing significantly as the seeding density was increased (Figure 2(a)). Highest basal CYP3A4 activity was observed at a seeding density of 75,000 cells per well, representing 144% of the basal activity observed at 50,000 cells per well and 215% of the basal activity observed at 25,000 cells per well (Figure 2(a)).

To establish the potential of each cell type for CYP3A4 induction, we treated cells with the prototypical CYP3A4 inducer rifampicin (10 *μ*M) for 72 hours and compared effects of this drug to DMSO-treated vehicle control samples (Figure 2(b)). In HepG2 cells, no statistically significant effects of rifampicin treatment were observed at any seeding density (Figure 2(b)). In contrast, CYP3A4 was potently induced by rifampicin in HepaRG cells at each seeding density. Compared to DMSO controls, rifampicin induced CYP3A4 activity 22-fold (*P* < 0.05) at cell seeding density of 25,000 per well and 33-fold and 31-fold (both *P* < 0.05) at seeding densities of 50,000 and 75,000 cells per well, respectively (Figure 2(b)). These data indicated firstly that the cryopreserved, differentiated form of HepaRG cells retains the high levels of expression and inducibility of CYP3A4 of the parental cell line [7] and secondly that CYP3A4 activity and inducibility in these cells are linked to cell seeding density, with the higher seeding densities of 50,000 and 75,000 cells per well having parity with regard to CYP3A4 fold induction.

**Figure 3:**
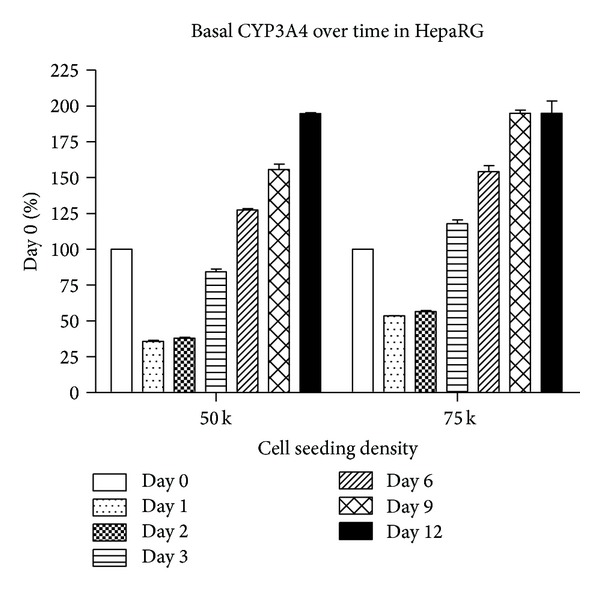
Basal CYP3A4 activity over time in culture. HepaRG cells were plated at 50,000 and 75,000 cells per well and CYP3A4 basal activity was measured over 12 days of continuous culture following thaw.

To further characterize the cryopreserved differentiated form of HepaRG cells as a model system and in particular to determine the suitability of these cells for long-term* in vitro* applications, we measured the basal CYP3A4 activity over a time period of 12 days after plating the cells for seeding densities of 50,000 and 75,000 cells per well (Figure 3). The cells showed high basal CYP3A4 activity in suspension immediately after thawing the cells on Day 0; however, at both seeding densities, CYP3A4 activity then decreased by approximately 50% during Days 1 and 2 in culture before recovering to Day 0 levels from Day 3 onwards (Figure 3). Basal CYP3A4 activity at each seeding density increased steadily over time from Day 3. By Day 12, basal CYP3A4 at both seeding densities stabilized at approximately 200% of Day 0 levels (Figure 3). These data indicate that the cryopreserved differentiated form of HepaRG is suitable for long-term* in vitro* studies and suggest that users ought to take account of the early declines in basal CYP3A4 activity during the first two days of plating into consideration when designing experiments. In all experiments in this study, we chose to wait until Day 3 before treating the cells with test chemicals.

**Figure 4:**
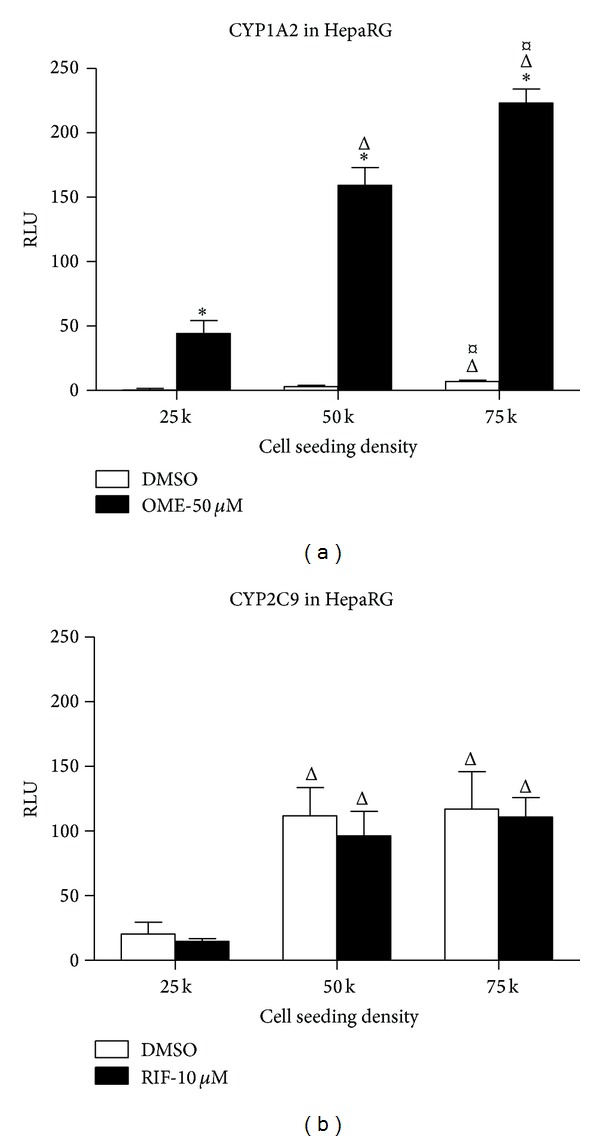
Induction of CYP1A2 but not CYP2C9 in cryopreserved HepaRG cells. (a) CYP1A2 induction in HepaRG cells plated in 96-well plates at seeding densities of 25,000, 50,000, and 75,000 cells per well and treated with DMSO (0.1%) or omeprazole (OME, 50 *μ*M) for 72 hrs starting at Day 3 in culture. Data represent mean ± SEM for 3 independent experiments. ∗ represents *P* < 0.05 versus DMSO-treated cells; Δ represents *P* < 0.05 versus cells seeded at 25,000 cells per well; *¤*  represents *P* < 0.05 versus cells seeded at 50,000 cells per well. (b) Treatment with DMSO (0.1%) or rifampicin (RIF, 10 *μ*M) for 72 hrs starting at Day 3 in culture in HepaRG cells plated in 96-well plates at seeding densities of 25,000, 50,000, and 75,000 cells per well. Data represent mean ± SEM for 3 independent experiments. Δ represents *P* < 0.05 versus cells seeded at 25,000 cells per well.

Having established that HepaRG cells express active CYP3A4, we then sought to examine activity of two other CYP isoforms known to be highly expressed in hepatocytes and involved in human drug metabolism, namely, CYP1A2 and CYP2C9 [23]. In HepG2 cells, no basal or induced activity of CYP1A2 or CYP2C9 was detectable. In HepaRG cells, under basal conditions, CYP1A2 activity was found to be weak but was found to be potently induced by treatment with 50 *μ*M omeprazole at each seeding density tested (Figure 4(a)). Although total induced activity of CYP1A2 was greatest at a seeding density of 75,000 cells per well, the greatest fold change over DMSO control was observed at a cell seeding density of 50,000 cells per well (53-fold increase; *P* < 0.05; Figure 4(a)). In contrast to CYP1A2, basal CYP2C9 activity was readily detectible in HepaRG cells, but no induction was observed using the prototypical inducer rifampicin (Figure 4(b)). Together, these data indicate that although the drug metabolizing capability of cryopreserved differentiated HepaRG cells is not fully equivalent to primary human hepatocytes, these cells express strongly inducible CYP3A4 and CYP1A2, and this CYP activity of HepaRG clearly distinguishes them from HepG2 cells. This feature of cryopreserved differentiated HepaRG is likely to be of great benefit for studies directly examining agents causing hepatotoxicity. For example, the activation of mycotoxin aflatoxin B1 is mediated in the liver by CYP3A4 and CYP1A2 [25], likely explaining the recent report that this agent is significantly more cytotoxic in cultures of HepaRG cells compared to HepG2 cells [26]. Clearly, the presence of active CYP isoforms in HepaRG cells confers significant advantages over nonmetabolically competent hepatocyte cellular models.

**Figure 5:**
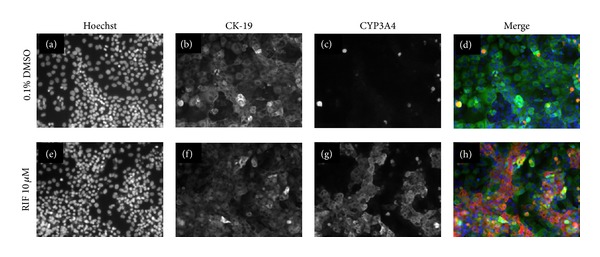
Multiplexed staining of cryopreserved HepaRG cells seeded at 50,000 cells per well and treated with DMSO vehicle control or 10 *μ*M rifampicin. (a) and (e): DMSO and rifampicin-treated cells, respectively, stained with Hoechst 33342 nuclear stain; (b) and (f): DMSO and rifampicin-treated cells, respectively, stained with anti-CK-19 antibody; (c) and (g): DMSO and rifampicin-treated cells, respectively, stained with anti-CYP3A4 antibody; (d) and (h): merged images of (a–c) and (e–g), respectively.

We next sought to characterize the utility of cryopreserved differentiated HepaRG cells for imaging-based High Content Analysis applications. Differentiated HepaRG cells stop proliferating and form colonies exhibiting a morphology resembling that of normal hepatocytes in primary culture with a dense cytoplasm and carboxy dichlorofluorescein diacetate- (CDFDA-) staining bile canaliculus-like structures surrounded by more flattened and clearer epithelial cells corresponding to biliary cells [27, 28]. We developed a multiplexed cell staining protocol to characterize the cryopreserved differentiated HepaRG cells used in this study, combining Hoechst 33342 dye to stain all cell nuclei within the cultures, an antibody against Cytokeratin 19 (CK-19), a marker of epithelial cells, and an antibody against CYP3A4. This staining approach enabled us to confirm the existence of both hepatocyte and biliary epithelial cells within cultures of cryopreserved differentiated HepaRG.  Figure 5 shows images of cells seeded at 50,000 cells per well, cultured for 3 days, then treated with DMSO vehicle control or 10 *μ*M rifampicin, and stained using the staining cocktail described. By examining images acquired from vehicle-treated cells (Figures 5(a)–5(d)), it becomes apparent that two distinct cell populations exist within the cultures, indicated by the presence or absence of CK-19 expression. Levels of CK-19 in cells expressing this marker are relatively homogeneous across the population (Figures 5(b) and 5(d)). In contrast, in vehicle-treated cells, a small population of cells stain strongly positive for CYP3A4, whilst the CYP3A4 protein is only weakly present in the remaining CK-19-negative cells (Figures 5(c) and 5(d)). We hypothesized that this weak CYP3A4 staining in vehicle-treated cells represented a basal level of CYP3A4 expression which may be upregulated by treatment of HepaRG cells with a CYP3A4 inducer. This was confirmed by examining images of rifampicin-treated HepaRG cells, where it was clearly observed that a dramatic upregulation of CYP3A4 expression occurred in response to this drug (Figures 5(g) and 5(h)).

**Figure 6:**
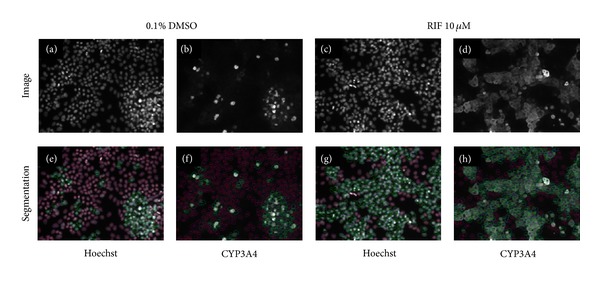
Segmentation of HepaRG cells for CYP3A4 expression using IN Cell Workstation software. This figure illustrates image analysis software tracing specified features of interest in representative images of HepaRG cells seeded at 50,000 cells per well. Upper panels (a–d) show staining images while lower panels (e–h) show image segmentation via HCA image analysis algorithms. Nuclear segmentation is shown by blue outlines in (e–h); cells expressing CYP3A4 are shown by green outlines; cells not expressing CYP3A4 are shown by red outlines. Hoechst nuclear stain is used to identify and count all cells in the images (a + e; c + g). Effects of 10 *μ*M rifampicin upon CYP3A4 expression versus vehicle control can be seen by comparing (d) with (b; h) and (f) shows the segmentation of CYP3A4 staining which enables subsequent quantitation.

Having performed qualitative assessment of images indicating that CYP3A4 induction by rifampicin is detectable by immunocytochemical methods, we then sought to determine if HCA image analysis software could be applied to quantify the visually observed effects of the drug. Through application of the multitarget algorithm within GE IN Cell Investigator software, we generated cell segmentation masks for Hoechst 33342 and CYP3A4 stains in vehicle and rifampicin-treated HepaRG cells (Figure 6). We found that, at the highest cell seeding density of 75,000 cells, the image analysis algorithm had difficulty segmenting individual cells due to their close proximity; however, no such issues were observed at seeding densities of 50,000 or 25,000. Within Figure 6, which shows cells seeded at 50,000 cells per well, it can be observed that, when properly optimized for the features of interest, the image analysis algorithm very accurately detects Hoechst stained nuclei (blue outlines), CYP3A4-positive cells (green outlines), and CYP3A4-negative cells (red outlines) (Figures 6(e)–6(h)). Accurate image segmentation is required to enable accurate quantitation of the features of interest within images. In this instance, we were particularly interested to examine the numbers of cells expressing CYP3A4, the intensity of the CYP3A4 staining, and features derived from the nuclear staining, such as cell count and nuclear size.

**Figure 7:**
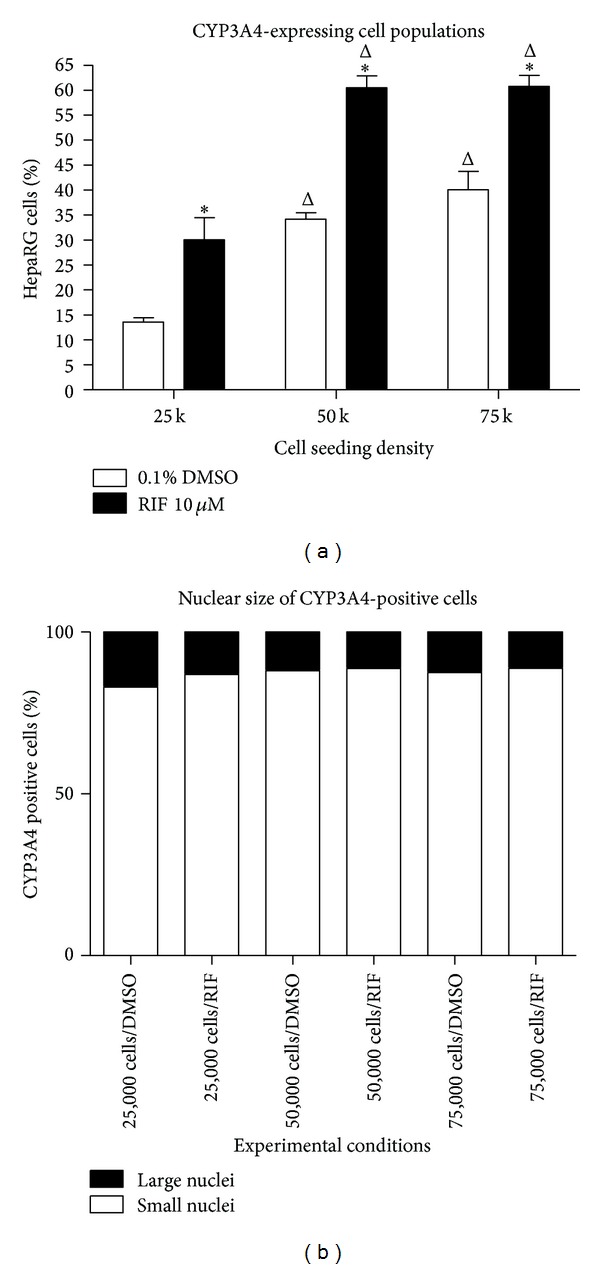
Quantification of numbers of CYP3A4-expressing cells via High Content Analysis. Using quantitative HCA image analysis, the percentage of HepaRG cells staining positively for CYP3A4 in vehicle and 10 *μ*M rifampicin-treated cells at each cell seeding density was determined. (a) shows the percentages of CYP-expressing cells within the entire HepaRG population for each condition. (b) shows the division of CYP3A4-expressing cells by nuclear size; small nuclei = ≤ 125 *μ*m^2^; large nuclei = > 125 *μ*m^2^. Data represent mean ± SEM for 3 independent experiments. ∗ represents *P* < 0.05 versus DMSO vehicle controls at the same cell seeding density; Δ represents *P* < 0.05 versus HepaRG cells seeded at 25,000 cells per well.

Quantitative image analysis data from the segmented HepaRG images is shown in Figure 7. We first quantified the percentage of CYP3A4 expressing cells relative to the total HepaRG cell population as assessed by automated counting of Hoechst-stained nuclei (Figure 7(a)). This analysis showed that HCA assessment of the percentage of CYP3A4-expressing cells data correlates well with the CYP3A4 basal and induced activity data generated using a luminescence-based P450 assay (Figure 2). In vehicle-treated cells, the percentage of CYP3A4-expressing cells as assessed by HCA was closely linked to cell density: at 25,000 cells per well, 14% of cells were CYP3A4-positive, increasing to 34% at 50,000 cells per well and 40% at 75,000 cells per well (Figure 7(a)). Rifampicin treatment significantly (*P* < 0.05) increased the percentage of CYP3A4-expressing cells at all seeding densities, to 30% at 25,000 cells per well, 60% at 50,000 cells per well, and 61% at 75,000 cells per well (Figure 7(a)). This novel approach provides evidence that High Content Analysis technology can be effectively employed to detect CYP3A4 in single cells* in situ*.

When reviewing the HepaRG staining images and cellular segmentation data, shown in Figures 5 and 6, it became apparent that the hepatocyte and biliary epithelial subpopulations within the HepaRG cultures have different-sized nuclei, with the hepatocyte population having smaller nuclei and the biliary epithelial cells having larger nuclei. Since the HCA analysis of Hoechst staining captured information on nuclear size, we were able to determine that the hepatocyte and biliary populations within HepaRG may be separated from one another during analysis by filtering the data based upon nuclear area. We created an image analysis filter applied at a nuclear area of 125 *μ*m^2^ so that cells with nuclei on either side of this filter could be analyzed separately and applied this to the data in Figure 7(a). Cells with nuclei with area lesser than 125 *μ*m^2^are hereafter referred to as “small nuclei” and those with nuclei greater than 125 *μ*m^2^ as “large nuclei.” We observed that, under each condition tested, the CYP3A4-expressing population of HepaRG cells is predominantly (85–90%) made up of small nuclei cells (Figure 7(b)). This suggests that analyzing only small nuclei cells within HepaRG cultures provides a greatly enriched population of hepatocytes for analysis and excludes the biliary epithelial cells. A small number of large nuclei, CYP3A4-expressing cells were also responsive to rifampicin (data not shown), indicating that these were also hepatocytes. Slight adjustments to the nuclear size filter may have slightly improved the detection percentage of the hepatocyte population, but our primary interest here was to exclude nonhepatocytes from analysis of hepatocyte-specific endpoints.

**Figure 8:**
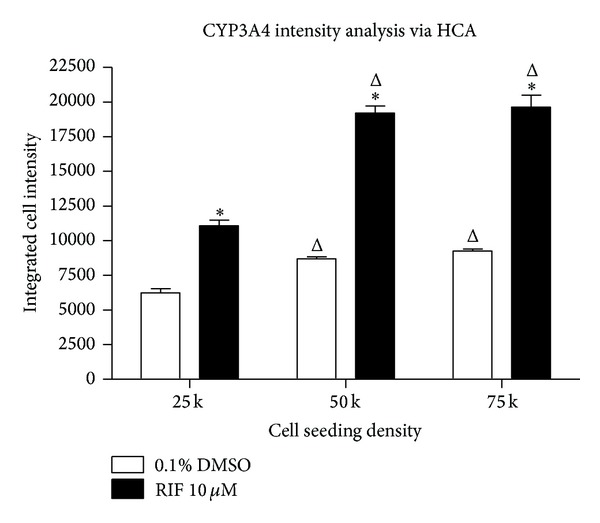
Quantification of CYP3A4 expression changes via HCA. Using quantitative HCA image analysis, the cellular intensity of CYP3A4 staining in small nuclei HepaRG cells treated with vehicle or 10 *μ*M rifampicin at each cell seeding density was determined. Data represent mean ± SEM for 3 independent experiments. ∗ represents *P* < 0.05 versus DMSO vehicle controls at the same cell seeding density; Δ represents *P* < 0.05 versus HepaRG cells seeded at 25,000 cells per well.

In addition to assessment of the numbers of CYP3A4 expressing cells, we also sought to determine if changes in CYP3A4 expression levels could be quantified via HCA.  Figure 8 shows analysis of the cellular intensity of CYP3A4 staining in small nuclei HepaRG cells. These data show that, in addition to quantifying numbers of CYP3A4-expressing cells, HCA analysis of CYP3A4 can be used to sensitively detect changes in expression levels as indicated by changes in the cellular fluorescence intensity. In vehicle-treated cells, the highest intensity of CYP3A4 expression was observed at a seeding density of 75,000 cells per well, representing 106% of the fluorescence intensity observed at 50,000 cells per well (not statistically significant) and 148% (*P* < 0.05) of that observed in cells seeded at 25,000 cells per well (Figure 8). Rifampicin treatment significantly (*P* < 0.05) increased the intensity of CYP3A4 expression at all seeding densities, evoking at 1.8-fold increase in cells seeded at 25,000 cells per well, a 2.2-fold increase at 50,000 cells per well, and a 2.1-fold increase at 75,000 cells per well (Figure 8). Thus, image analysis-based techniques can be used to effectively generate sensitive, quantitative, and cell-by-cell data on CYP3A4 from intact HepaRG cells* in situ*. Based on these data, we have found 50,000 cells per well to be the optimal seeding density for HCA assessment of HepaRG in 96-well plates.

There are significant advantages to be derived from our observation that it is possible to algorithmically separate the HepaRG hepatocyte population from the biliary epithelial population during image analysis. Firstly, as with other heterogeneous or cocultured cell populations, it is imperative to be able to study cell-specific functions in isolation, so as not to contaminate cell-specific data with data from other cells residing within the culture milieu. This is a limitation of assays which rely on whole well analysis or cell lysis steps during sample preparation. Image analysis renders it possible to study individual cell types* in situ* without the need to disrupt the culture and/or physically sort cells. High Content Analysis of cellular images is known to be a powerful technique for isolating different cell populations within complex cultures [29]; however, this approach generally relies on inclusion of cell-type-specific markers in order to segment each population separately. As most automated microscopes and HCA instruments have a limited number of fluorescence channels (typically 3-4) available per assay, multiple channels are frequently used simply for cell-specific identification markers and the requisite nuclear stain and thus are not available to study other cellular events. Indeed, when commencing this study, we expected that inclusion of hepatocyte-specific (CYP3A4 or perhaps HNF4*α*) and epithelial-specific (CK-19) markers would be required to separate the subpopulations of hepatocyte and biliary epithelium cells within HepaRG cultures during analysis. However, the differences in nuclear size between these populations observed and reported here render these additional stains unnecessary, as nuclear size can be used to effectively separate these populations. This saves both time and resources and frees up fluorescent imaging channels to examine other features of interest within the cells.

**Figure 9:**
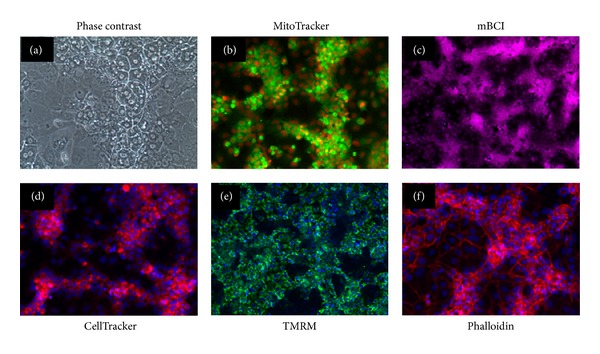
Labeling of HepaRG with cell function and structure dyes. Images shown represent HepaRG cells plated on 96-well collagen coated plates in growth media at 50,000 cells per well and cultured for 3 days. Images shown represent label-free phase contrast imaging of live cells (a); live cells stained with MitoTracker Green FM (b); live cells stained with Monochlorobimane (mBCI) (c); live cells stained with CellTracker Red CMTPX (d); live cells stained with Tetramethylrhodamine, Methyl Ester, and Perchlorate (TMRM) (e); and fixed cells stained with Alexa Fluor 568 Phalloidin (f).

As proof of concept for other imaging-based assessments that could be performed in cryopreserved differentiated HepaRG cells and multiplexed with CYP3A4 expression assays, we performed preliminary imaging staining experiments in HepaRG, using a variety of cell function and cell tracing reagents relevant to hepatotoxicity (Figure 9). Figure 9(a) shows a phase contrast image of live HepaRG cells. As image analysis algorithms for phase contrast and bright field images improve and time-lapse imaging becomes more accessible, quantitative, label-free cell health assays will likely see an increase in usage. Figure 9(b) shows MitoTracker Green FM staining in live HepaRG cells; this dye localizes to mitochondria regardless of mitochondrial membrane potential and can be used to determine changes in mitochondrial mass. Figure 9(c) shows Monochlorobimane (mBCI) imaging in live HepaRG cells; mBCI is nonfluorescent but forms a stable, fluorescent adduct with glutathione (GSH). The fluorescent signal recorded over time is directly proportional to the concentration of GSH and serves as a useful indicator of oxidative stress. Figure 9(d) shows CellTracker Red CMTPX staining in live HepaRG cells; this dye is useful for long-term cell tracing experiments. Figure 9(e) shows live HepaRG cells stained with Tetramethylrhodamine, Methyl Ester, Perchlorate (TMRM); this dye is widely used to detect changes in mitochondrial membrane permeability in hepatocytes. Finally, Figure 9(f) shows Alexa Fluor 568 Phalloidin staining in fixed HepaRG cells; this can be used to measure cytoskeletal changes and is widely used as a component of cell viability assays.

Based on the data presented here, we believe it is now possible, using the culture and assay conditions established in this study, to perform many more multiplexed imaging-based assays using HepaRG cells, having confidence that metabolic competence is maintained and the hepatocyte subpopulation can be analyzed via quantitative imaging techniques. A plethora of potential multiplexing reagents are available to detect other features of interest, for example, cell death, cell signaling molecules, and functional markers of mechanisms of hepatotoxicity in HepaRG cells, and HCA provides a high-throughput format suitable for large-scale drug and chemical safety screening. Looking ahead, more dynamic live cell analysis-based applications for hepatotoxicity seem likely to emerge, perhaps utilizing novel microfluidic devices [30]. We expect that in future HCA-compatible live cell substrates for CYP3A4 and other drug metabolism enzymes can be combined with fluorescent biosensors and novel devices for dynamic live hepatocyte cultures in order to shed further light upon the kinetics of drug metabolism and the cellular events underpinning hepatotoxicity.

## 5. Conclusions

In recent years, powerful automated microscopes and automated image analysis algorithms have become increasingly accessible to more scientists, and these are enabling exciting novel research to be carried out upon individual cells* in situ*, as opposed to population-based analyses of harvested cells. In this study, we have demonstrated the feasibility of an automated imaging-based approach for assessing CYP3A4 expression and activity using metabolically competent HepaRG cells. This study has (a) demonstrated that cryopreserved differentiated HepaRG cells retain key functional hepatocyte characteristics, notably maintaining inducible CYP3A4 activity over time, (b) shown that these cells may be employed for multiparametric High Content Analysis applications, and (c) provided information on optimal cell culture and image analysis conditions for the application of these cells to imaging-based CYP3A4 expression studies and multiparametric hepatotoxicity assessment.

Interest in imaging-based assessment of cellular toxicity continues to grow, and hepatotoxicity is of particular interest because of its implications for the pharmaceutical industry. Viable alternative cellular models to primary human hepatocytes and nonrepresentative hepatoma cell lines like HepG2 have long been sought. In this study, we have demonstrated that the cryopreserved differentiated form of HepaRG retains many functional features of human hepatocytes and is highly amenable to quantitative imaging-based assessment of hepatocyte-specific endpoints under conditions where strong and stable CYP3A4 activity is maintained. As imaging-based hepatotoxicity assessment applications continue to emerge, cryopreserved differentiated HepaRG cells will likely be increasingly employed as metabolically competent surrogates for primary human hepatocytes.
